# Design and Evaluation of Tretinoin Fatty Acid Vesicles for the Topical Treatment of Psoriasis

**DOI:** 10.3390/molecules28237868

**Published:** 2023-11-30

**Authors:** Yaxin Zhao, Chao Wang, Bohang Zou, Lin Fu, Shushan Ren, Xiangyu Zhang

**Affiliations:** College of Pharmacy, Jiamusi University, Jiamusi 154007, China; 218053053@stu.jmsu.edu.cn (Y.Z.); wangchao0824@outlook.com (C.W.); zoubohang@outlook.com (B.Z.); fulin112233@outlook.com (L.F.); renshushan@outlook.com (S.R.)

**Keywords:** Tretinoin, fatty acid vesicles, film hydration method, anti-psoriasis

## Abstract

The goal of the current study was to explore the potential benefits of Tretinoin (Tre) fatty acid vesicles (Tre-FAV) as a prospective antipsoriatic topical delivery system. This promising system can counteract the drug challenges in terms of its extremely low aqueous solubility, instability, skin irritation, and serious systemic adverse effects. Tre-loaded fatty acid vesicles were successfully developed and entirely characterised. The selected formulation was investigated for in vitro release, ex vivo skin retention and psoriasis efficacy studies. The characterisation results of Tre-FAV showed it has a globular shape with a particle size of 126.37 ± 1.290 nm (0.188 ± 0.019 PDI). The entrapment efficiency and zeta potential were discovered to be 84.26 ± 0.816% and −28.9 ± 1.92 mV, respectively. Encapsulation of the drug in the fatty acid vesicles was also strengthened by differential scanning calorimetric and powder FTIR diffraction studies. In vitro release results showed that Tre-FAV significantly increased skin absorption and retention in comparison to the Tre solution. The topical application of Tre-FAV to a mouse model confirmed that it has superior in vivo antipsoriatic properties in terms of well-demarcated papules, erythema and reduced epidermal thickness in comparison to other treatments. The weight of the spleen and the levels of the cytokines IL-17 and IL-6 decreased after treatment. In conclusion, FAV dramatically increased the water solubility and skin permeability of Tre and its anti-psoriasis activity.

## 1. Introduction

Psoriasis is an autoimmune-mediated inflammatory skin disease characterised by erythematous, well-demarcated papules, hyperproliferatic keratinocytes and intense leukocyte involvement with cutaneous inflammation [1,2,3,4,5]. It is estimated to affect 2–3% of the patient population around the world [6,7,8]. Psoriasis vulgaris accounts for over 90% of all cases and is the most prevalent type [9]. Its treatment options include both percutaneous topical treatment and systemic therapies. Percutaneous topical therapy is a route of administration in which a drug is given through the skin for local purposes. It is believed that most of the triggers of skin disease are to be found under the skin, and locally delivered medication is favoured over more systemic approaches. At current levels, topical therapy is the cornerstone of treatment for mild to moderate-to-severe psoriasis and is often the first-line and complementary treatment for more severe psoriasis [10,11]. Traditional treatment usually involves applying anti-psoriasis medication in the form of ointments, creams or lotions directly to the diseased skin tissue, with the frequency of application varying depending on the severity of the inflammation, averaging 1–2 times per day. These topical formulations are limited in their ability to deliver the drug across the stratum corneum, which is critical for the permeation of the drug into the basal layer of the epidermis to exert its pharmacological effects [12]. However, the topical delivery of these products remains unsatisfactory due to inadequate drug penetration and patient compliance [13]. As such, there is a significant need to explore new potent topical medications with therapeutic efficacy for individuals with psoriasis.

Tretinoin (Tre) is used topically to treat various skin conditions. These include acne vulgaris, ichthyosis and psoriasis [14,15,16]. The therapeutic potential of Tre in psoriasis is partly due to its mechanism of action in regulating skin cell growth and maturation, sebum synthesis and collagen production by virtue of its ability to bind to retinoic acid transport receptors. Unfortunately, oral Tre is unacceptable because of the serious side effects associated with taking retinoids, such as teratogenicity, CNS symptoms, etc. [17,18]. The topical presentation of Tretinoin allows for the provision of the effective treatment of dermatological conditions while at the same time reducing systemic overload and toxicity [19]. Nevertheless, its application in a topical form is hindered by several imperfections, including cutaneous irritation, extremely weak water permeability and high sensitivity to light [14].

To address the problems mentioned in the previous paragraph, a new dosage form for topical administration is necessary. Vesicular structures have the capacity to deliver the drug in a targeted manner while forming a local reservoir in the skin, enhancing the potential for transdermal delivery. The potency of these vesicle systems is a function of the composition of the vesicles and the method of preparation, as these affect the physico-chemical behaviour of the vesicles. Fatty acid vesicles (FAVs) are closed binary-layer aggregates formed by fatty acids and their ionic compound self-assembly. In addition, due to their high solubility, FAVs ensure excellent separation in various biological membranes [20,21]. Fatty acids are abundant in the skin, which help to strengthen the skin’s barrier strength and can be employed as an effective skin-penetration enhancer [22,23].

The objective of the work reported in this study was to encapsulate the poorly soluble drug Tre in FAV-based pharmaceutical formulations through simple approaches of aqueous hydration and to investigate the effect of fatty acid vesicles on the encapsulation of Tre and its release in vitro. An in vitro transdermal study was conducted to determine their transdermal penetration properties. An in vivo pharmacodynamic study was conducted to evaluate their anti-psoriasis effect. These vesicles have the potential to improve the bioavailability of the anti-psoriasis drug, provide appropriate transdermal penetration properties and have broad implications for biomedical applications.

## 2. Results and Discussion

### 2.1. Morphology of Vesicles

**Figure 1:**
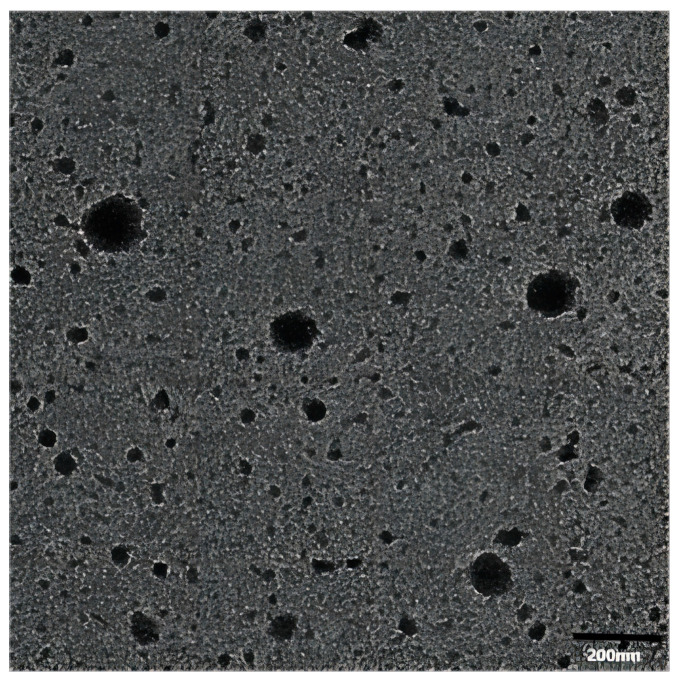
TEM image of Tre-FAV.

Transmission electron microscopy (TEM) was conducted to evaluate the shape and surface morphology of the drug-loaded vesicles. Figure 1 illustrates a TEM image of the vesicles showing their nearly spherical shape with an average size of 150 nm. The oleic vesicles with this size range meet the requirement as a topically delivery system for clinical applications.

### 2.2. The Size, Zeta Potential and Polydispersity Index (PDI) of Vesicles

**Figure 2:**
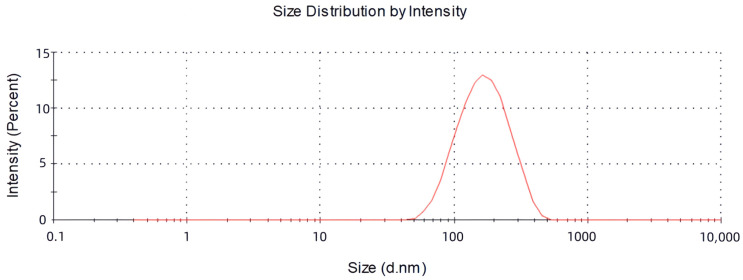
Particle size distribution of Tre-FAV.

**Figure 3:**
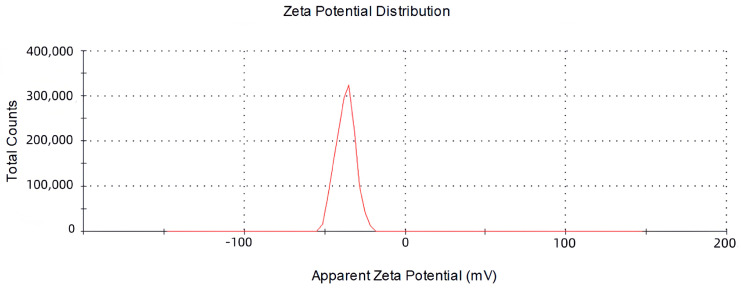
Zeta potentials of Tre-FAV.

The objective of this study was to develop the percutaneous delivery vesicles of oleic acid using Tre acid as carriers. Drug-loaded vesicles were optimised regarding the vesicle size, drug encapsulation, zeta potential and polydispersity index (PDI). The maximum encapsulation rate of Tre was (84.26 ± 0.816%), while the ratio of drug to oleic acid was 4:6. The experiments demonstrated that the loading of oleic acid vesicles with the drug was related to the proportion of oleic acid to the drug. The decrease in encapsulation efficiency may be due to saturation of the drug molecules in the bilayer structure and destabilisation of the vesicular membrane as a result of continuous drug application [24,25]. The measured mean particle size was (126.37 ± 1.290) nm, the PDI was (0.188 ± 0.019) and the zeta potential was (−28.9 ± 1.92) mV, as described in Figure 2 and Figure 3. Tre-FAV has a uniform distribution of particle size, negative zeta potential and absolute value greater than 25 mV, so there is strong electrostatic repulsion between vesicles, as well as strong stability.

### 2.3. Differential Scanning Calorimetry (DSC)

**Figure 4:**
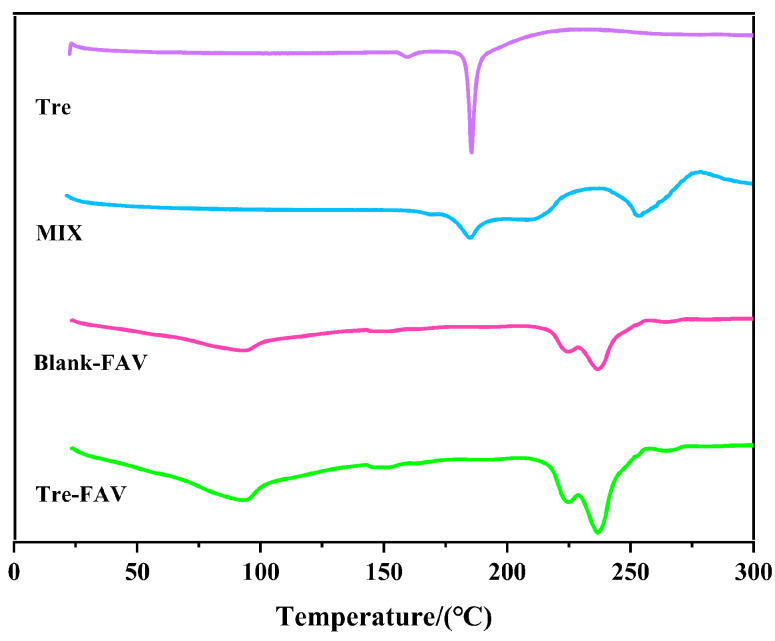
Differential scanning for thermal analysis.

DSC is a method of investigating the melting and recrystallisation behaviour of crystalline materials, which include FAV. Pure Tre, Tre-FAV, blank FAV and physical mixtures were subject to DSC investigation to evaluate their thermal behaviours. Figure 4 shows four types of thermograms. Thermograms of Tre exhibited sharp endothermic peaks at 185.60 °C. For the physical mixtures, the thermogram peaks of the excipients appeared close to 250 °C, and the thermogram peaks of Tre were still present in the spectra. Conversely, the heat absorption peak of Tre vanishes in the Tre-FAV and blank FAV spectra, which is an indication of the encapsulation of drug molecules within the fatty acid shell.

### 2.4. Fourier Transform Infrared Spectrometer (FTIR)

**Figure 5:**
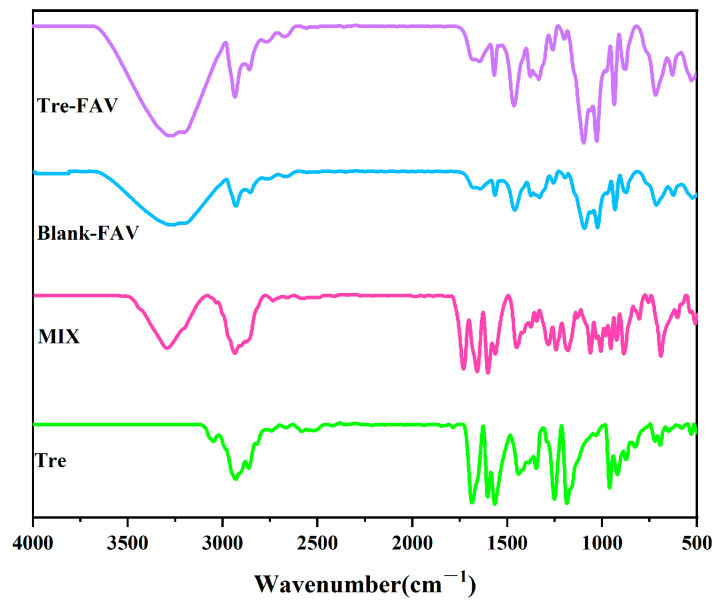
Fourier infrared spectroanalysis.

Figure 5 shows the presence of Tre peaks at 1689.62, 1602.82 and 1249.85 cm^−1^, and in physical mixtures, the infrared spectra still show the characteristic peaks of Tre. However, in the Tre-FAV and blank FAV spectra, the characteristic peaks of Tre vanish, which indicates that there have been no interactions introduced between the drug and excipients, further suggesting that Tre drug is encapsulated in the vesicles.

### 2.5. Stability Studies of Vesicles

The study was intended to investigate the influence of storage at differing temperatures on the stability of Tre-FAV. When stored at 25 °C for 1, 7, 15 and 30 days, Tre-FAV showed no significant changes over the first 15 days, with a small amount of yellow precipitate at the bottom after 30 days, probably leaked Tre. When stored at 4 °C for 1, 7, 15 and 30 days, the appearance of the solution remained consistent, with the solution maintaining homogeneity and stability. Table 1 and Table 2 show the average particle size, zeta potential and encapsulation efficiency. The slight decrease in the encapsulation rate, trivial increase in particle size and unimportant change in potential indicated that Tre-FAV exhibits exceptional storage stability performance.

### 2.6. In Vitro Transdermal Studies

#### 2.6.1. In Vitro Transdermal Permeation Studies of Vesicular Dispersion

**Figure 6:**
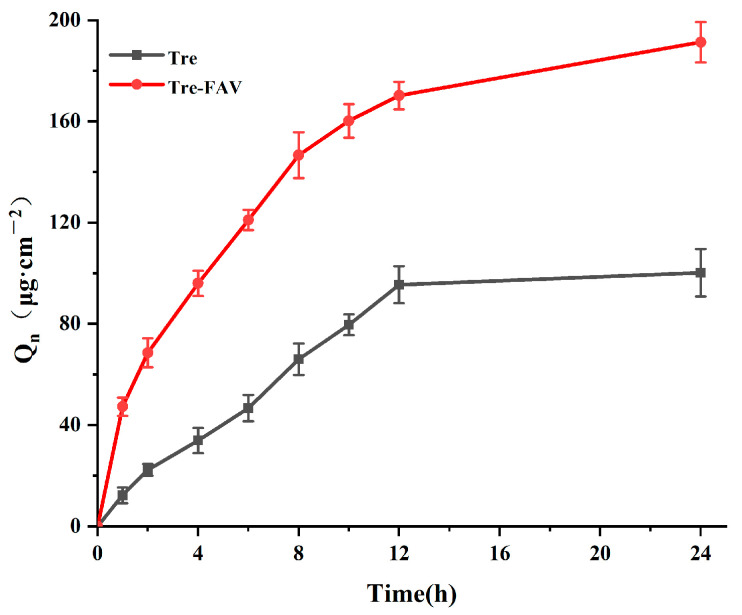
Transdermal cumulative curve of Tre solution and Tre-FAV (*n* = 3).

The Franz diffusion method was used for the drug transdermal permeation study, and the results are presented in Figure 6. It is clear from the graphs that the trends in permeation of the Tre solution and Tre-FAV are basically the same, although the cumulative permeation of fatty acid vesicles is higher for the Tre solution. Likewise, in the ex-vivo permeation model, Tre-FAV demonstrated a 2.7-fold improvement in drug permeation (92 μg/cm^2^) over that of Tre solution (34 μg/cm^2^). Therefore, fatty acid vesicles improve the skin permeation rate of Tre solution to different degrees, which shows that Tre solution has difficulty achieving the ideal effective concentration for transdermal administration and requires preparation as fatty acid vesicles to promote its transdermal penetration.

#### 2.6.2. Skin Retention of Vesicular Dispersion

**Figure 7:**
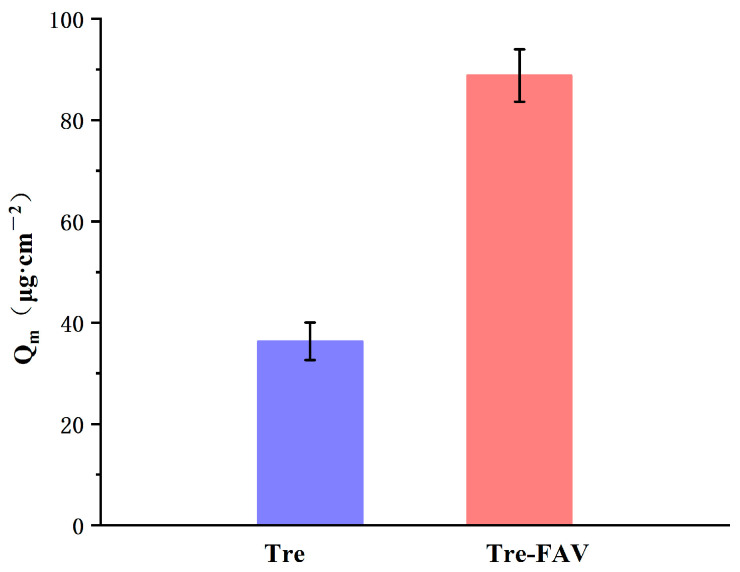
Skin retention of Tre solution and Tre-FAV (*n* = 3).

The results of the in vitro skin retention study are presented in Figure 7. The 24 h skin retention for Tre-FAV was 88.79 μg/cm^2^, and the Tre solution was 36.34 μg/cm^2^. Tre-FAV was 2.89 times higher than that of the Tre solution, mainly because fatty acids are very soluble and can easily and rapidly break down into artificial and natural membranes [20]. Accordingly, FAV has better skin-penetration behaviour.

### 2.7. In-Vivo Pharmacodynamic Study

#### 2.7.1. Establishment of Psoriatic Animal Models

**Figure 8:**
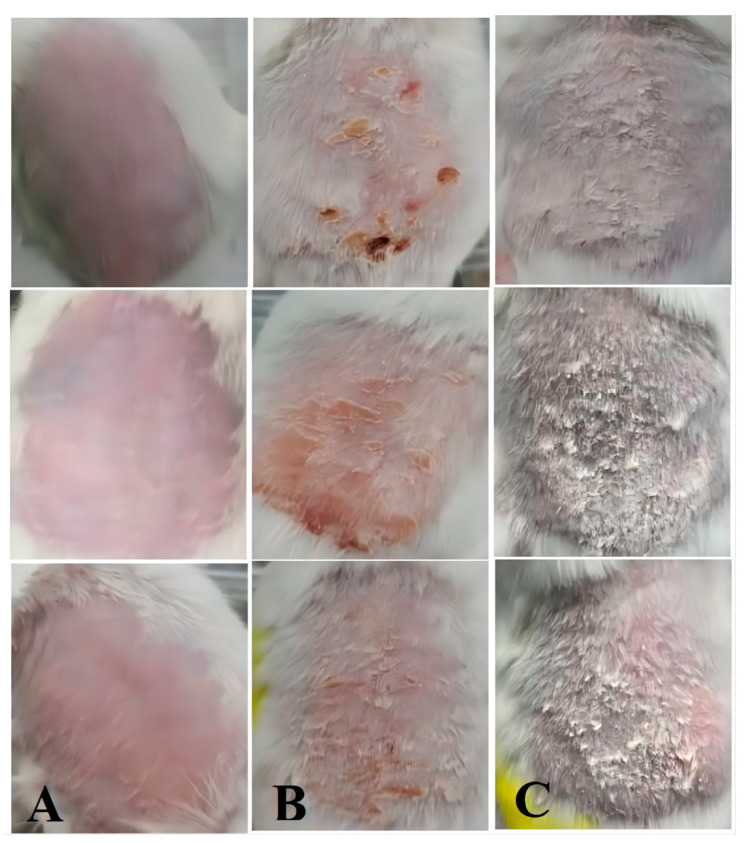
Skin changes in the mouse model. Skin of normal mice (**A**), skin state of mice on the third day of modelling (**B**) and skin state of mice on the seventh day of modelling (**C**).

IMQ is a powerful immune stimulator and is a TLR 7/8 ligand. It has been involved in both inducing and exacerbating psoriasis. During the preparation of the model mice, it was observed that imiquimod resulted in skin folds, erythema and fine scales on the back skin of mice within 2–3 days of continuous topical application, after which, with a longer induction time, the skin changed from light pink to dark red and thickened significantly and the amount of surface scales increased [26]. The most significant skin changes were observed when the drug was continuously administered for 6–7 days, as indicated in Figure 8.

#### 2.7.2. Psoriasis Area Severity Index (PASI) Evaluation

**Figure 9:**
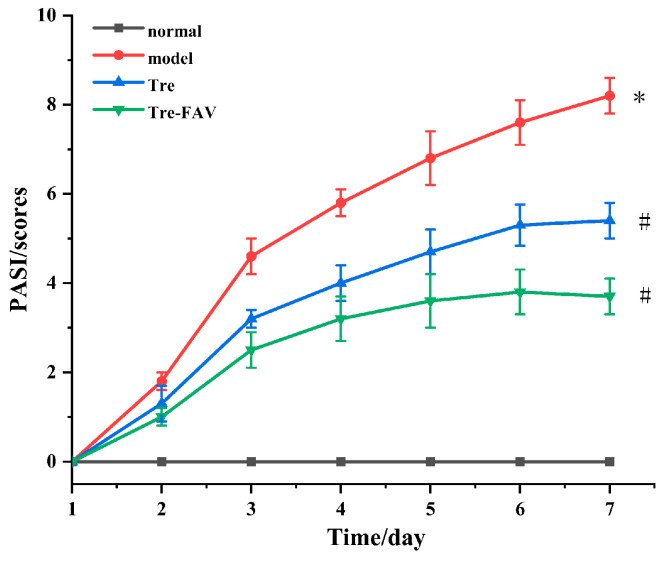
Changes in back skin PASI score in each group (*n* = 3, Comparison with blank groups, * *p* < 0.05; Comparison with model groups, # *p* < 0.05).

PASI scoring has been recognised as a preliminary qualitative assessment in regards to disease progression, as well as treatment responses, in psoriasis therapy [27]. The erythema and scaling of the skin was assessed throughout the groups and the results of this evaluation are presented in Figure 9. Back erythema, scales and skin thickening gradually developed in the model group. Mice had significantly reduced erythema after treatment in each prescription group. A consistent progression of erythema, scales and skin thickening were observed during the experiment. The severity of the three parameters decreased differently in different treatment groups.

#### 2.7.3. Histopathology

**Figure 10:**
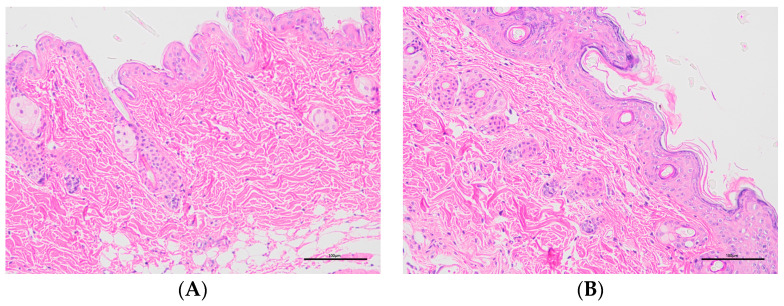

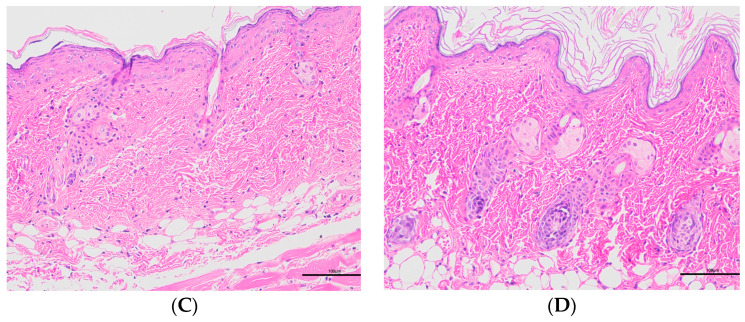
Histopathological observation of skin lesions of mice in each group (HE × 200). Normal mouse tissue section observation (**A**), model mouse tissue section observation (**B**), Tre solution treatment group section observation (**C**) and Tre-FAV treatment group mouse tissue section observation (**D**).

Histopathology was performed on the collected skin samples. Figure 10 shows the histopathological images of different groups. Histopathology sections showed that the epidermal layers of the skin of normal mice were thin and composed of one to two layers of epidermal cells (Figure 10A) [28,29,30]. In contrast to the normal group, the model group exhibited the abundant abnormal proliferation of SC cells and poor keratinisation, resulting in severe parakeratosis (Figure 10B). The data demonstrated that the skin structure was intact and normal for the Tre-FAV and Tre solution (Figure 10C,D). In the Tre-FAV group, there was a thickening of the stratum corneum, but it was not obvious, and the number of infiltrating inflammatory cells was also much less than that of the model group. This indicates that the prepared Tre-FAV has a good anti-psoriasis effect on psoriasis.

#### 2.7.4. Weight Ratio of Spleen to Body (Spleen/Body wt%)

**Figure 11:**
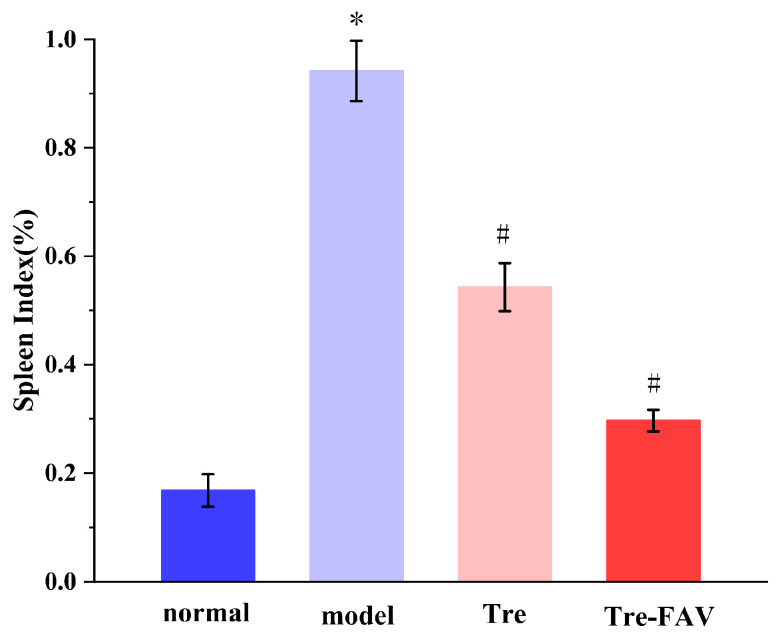
Splenic index of each group (*n* = 3, Comparison with blank groups, * *p* < 0.05; Comparison with model groups, # *p* < 0.05).

In Figure 11, the mean spleen/weight ratio increased in the model group compared to the blank control group. In comparison with the model group, both Tre-FAV-treated groups had a significant decrease in the spleen weight ratio (*p* < 0.05), the mass of the Tre-FAV group increased on days 5 and 6, spleen size and quality decreased and the spleen index decreased (*p* < 0.05) [31,32].

#### 2.7.5. The Levels of IL-17 and IL-6 Detection

**Figure 12:**
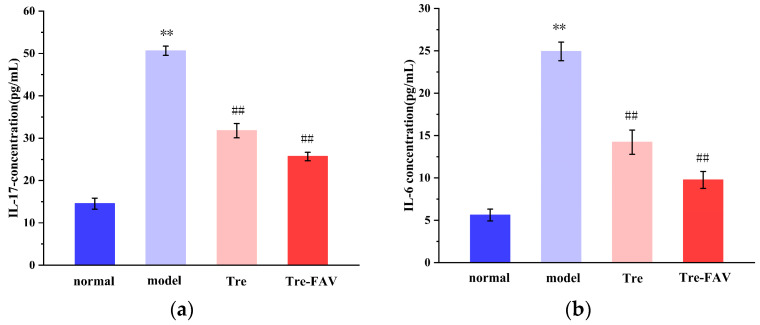
The expression of (**b**) IL-6 and (**a**) IL-17 in mice with psoriasis (*n* = 3, Comparison with blank groups, ** *p* < 0.01; Comparison with model groups, ## *p* < 0.01).

The cytokines IL-17 and IL-6 play a key role in the pathophysiology that leads to psoriasis [33,34]. Figure 12 demonstrates that the serum concentrations of IL-17 and IL-6 in the experimental group compared to the blank control group were significantly elevated (*p* < 0.01). Compared to the control group, IL-17 and IL-6 levels were significantly reduced in each treatment group. The highest anti-psoriatic potency of Tre-FAV could be the result of its improved ability to reduce the levels of the cytokines IL-17 and IL-6.

### 2.8. Discussion

The research demonstrated that Tre-loaded oleic acid vesicles could be successfully manufactured and that the drug, once loaded into oleic acid vesicles, could be effectively delivered via the transdermal route. The FAV-loaded Tre is prepared using thin film hydration and the optimised formulation has a small particle size and PDI to improve permeation through the skin. The vesicle potential is high, indicating strong electrostatic repulsion and stability within the vesicle. The results of stability experiments showed that the carriers maintained good stability at both 4 °C and 25 °C. The results of in vitro transdermal experiments showed that FAV significantly increased the amount of drug penetrating the skin surface and improved the retention of the drug, which demonstrates its efficacy in the long-term treatment of psoriasis in local lesions. The mouse model of IMQ-induced psoriasis is a conventional model, employing a straightforward and cost-effective evaluation method. Based on the psoriasis PASI score and skin histopathology samples, Tre-FAV reduced mouse skin inflammation, and the ELISA kit showed that levels of IL-6 and IL-17 were reduced in mice. The results of the splenic index demonstrate that FAV can efficiently reduce mouse spleen enlargement, reduce the frequency of autoimmune reactions and provide notable therapeutic benefits to psoriasis-inflicted mice. This provides evidence that Tre-FAV is more effective in treating psoriasis and yields a satisfactory therapeutic outcome, and these observations indicate that Tre-loaded FAV may constitute a promising antipsoriatic therapy.

## 3. Materials and Methods

### 3.1. Materials

Oleic Acid (OA) was purchased from Sinopharm (Beijing, China). Tretinoin (Tre) were purchased from Aladdin Industrial Corporation (Shanghai, China). Methanol and sodium hydroxide were supplied by Tianjin Kaitong Chemical Reagent Factory (Tianjin, China). PBS (pH 7.4) was supplied by Beijing Solarbio Science Technology Co., Ltd. (Beijing, China). Imiquimod cream (IMQ; 5%) was purchased from Sichuan Mingxin Pharmaceutical (Sichuan, China). Bai Vaseline was purchased from Macklin (Shanghai, China). All other reagents were analytical grade, and all experiments used double-distilled water.

### 3.2. Animals

KM mice, aged 2- to 8-weeks-old, were purchased from Changchun Changsheng Biotechnology Co., Ltd. (Changchun, China). The in vivo experiments were performed according to the guidelines of the Experimental Animal Administrative Committee of Jiamusi University. All institutional and national guidelines for the care and use of laboratory animals were followed.

### 3.3. Preparation of Vesicular Formulations

The thin film hydration method has been utilised to prepare Tre-loaded vesicles [35,36]. For thin film manufacture, the drug and oleic acid were melted in a methanolic round bottom flask with the solvent being evaporated (Shanghai Shensheng Technology Co., Ltd., Shanghai, China) through a rotary evaporator under vacuum conditions. The organic solvent was completely evaporated, leading to the production of a dried film, which was stored overnight to remove any residual traces of methanol and to preclude the formation of emulsions as a result of residual amounts of methanol. For two hours at room temperature, the film was hydrated with a phosphate buffer (pH 7.4). The vesicular suspension that formed as a consequence of hydration was ultrasonicated to form uniformly sized vesicles. The formulations were optimised by modulating the ratio of oleic acid to drug.

### 3.4. Characterisation of Oleic Acid Vesicular Formulations

#### 3.4.1. Morphology of Vesicles

The shape and surface morphology of drug-loaded oleic acid vesicles were assessed via transmission electron microscopy (TEM) (JEOL JEM-1010, Tokyo, Japan) [37]. A sprinkling of the sample was applied to a copper grid with a carbon coating. The film was then counterstained with 1% phosphotungstic acid and dried on the grid. Subsequently, the lattice was air-dried before the samples were evaluated via TEM.

#### 3.4.2. The Size, Zeta Potential and Polydispersity Index (PDI) of Vesicles

The size fraction and zeta potential of FAV were determined with the aid of a Zetasizer Nano-Series (Shimadzu Instruments Co., Suzhou, China). The Tre-FAV suspension was diluted 200 times and measured at room temperature using a Malvin nanometer, with all measurements performed in triplicate [38].

#### 3.4.3. Encapsulation Efficiency

Here, 5 mL of the prepared Tre-FAV solution was placed into a 10 mL centrifuge tube with a pipette gun. And the centrifuge was set at 3000 rpm for 10 min. Then, 1 mL of supernatant was taken out and mixed with 1M NaOH into a 10 mL brown volumetric bottle, and it was subjected to 10 min of ultrasound. Another 1 mL Tre-FAV solution was added to a 10 mL brown volumetric bottle, 1M NaOH was used as demulsifier and the solution was demulsified via ultrasound for 10 min after obtaining a constant volume [39]. The yield (Y%) of FAV was determined by using an Agilent 1200 series HPLC-DAD system (Agilent Technologies, Santa Clara, CA, USA). An AC 18 column (Agilent Zorbax; 4.6 mm × 250 mm, 5 μm) with a guard column was utilised for separation at room temperature. The mobile phase was methanol–0.4% H_3_PO_4_ solution (91:9, *v*/*v*) and the elution rate was 1 mL/min. All samples were added to the column in a volume of 10 μL. The wavelength of detection for Tre was 350 nm, which was calculated as the drug content in vesicles (m_1_), and the drug dosage was calculated as the total drug dose (m_2_). The encapsulation efficiency (EE) of Tre-FAV was calculated according to the following Formula (1)

(1) $$\mathrm{EE}\%=\frac{m_{1}}{m_{2}}\;\times 100\%$$

#### 3.4.4. Differential Scanning Calorimetry (DSC)

The thermal behaviour of the resulting beads was investigated by using a differential scanning calorimeter (STA409PC, Beijing Hengjiu Experimental Equipment Co., Ltd., Beijing, China) [24]. The pure Tre, Tre-FAV, blank FAV and physical mixture material samples were taken separately. For the study, the sample was placed in an aluminium dish and then heated from 25 to 300 °C at a rate of 5 °C/min with a surrounding nitrogen flow at a rate of 20 mL/min. An empty pan of a similar size was used as a reference.

#### 3.4.5. Fourier Transform Infrared Spectrometer (FTIR)

The Infrared Spectroscopy (Spectrum100, PerkinElmer Company, Waltham, MA, USA) study was used to find out the chemical stability of the drug during formulation [25]. The lyophilised vesicles were sampled and analysed for this study using IR spectroscopy in the 4000 cm^−1^ to 500 cm^−1^ range to compare pure Tre, Tre-FAV, blank FAV and a physical mixture of the ingredients.

#### 3.4.6. Stability Studies of Vesicles

It is generally recognised that product stability can be quantified as the ability of a given formulation to preserve its physical, chemical, therapeutic and toxicological properties. The optimised vesicles were placed in transparent glass bottles, tightly covered with aluminium foil and stored at 4 °C and 25 °C. Samples were taken at 1, 7, 15 and 30 days to determine stability parameters, such as the particle size, PDI and zeta [40].

### 3.5. In Vitro Transdermal Study

#### 3.5.1. Processing of the Isolated Mouse Skin

The first step was to clip the fur on the backs of the mice. Next, a cotton ball was dipped into depilatory cream and applied as a thin layer to the desired area. After 2–3 min, the cream was washed off with lukewarm water and the area was dried with gauze; then, the mice were killed 12 h after hair removal. A blunt dissection was performed to remove the full thickness of the dorsal skin, and the skin was stored at −20 °C. 

#### 3.5.2. Ex Vivo Skin Permeation Study

The skin permeability of vesicles was measured using a YB-P6 Franz diffusion cell (Tianjin Pharmacopoeia standard Instrument Factory, Tianjin, China) [41]. After thawing at room temperature, the skin was positioned at the interface between the donor and recipient chambers, with the SC directed towards the donor. The integrity of the skin membranes had been checked prior to application of the formulation in the donor compartment. The diffusion area accessible from the mouse skin was 1.766 cm^2^. The receptor chamber was primed with 14 mL of phosphate buffered saline (pH 7.4), maintained at 37 ± 0.5 °C and agitated using a magnetic bar at 300 rpm. Subsequently, 1 mL of Tre-FAV and Tre solution was added to the donor compartment. Over a period of 24 h, 1 mL samples were taken at various time intervals of 1, 2, 4, 6, 8 12 and 24 h. Samples from the receptor partition (1 mL) were withdrawn periodically and analysed for drug content via HPLC. The 1 mL samples were then exchanged for the same volume of phosphate buffer, pH 7.4, kept at 37 ± 0.5 °C. After filtration using a 0.22 μm microporous filter, the cumulative transmittance Qt of skin per unit area at each time point was calculated using the following Formula (2). All experiments were performed in triplicate.

(2) $$Q_{n}={\frac{V_{0}\times C_{n}+\sum_{i=1}^{n-1}C_{i}\times V_{i}}{A}}_{}$$

#### 3.5.3. In Vitro Skin Retention Experiments

At the end of the permeation experiment, the retention of Tre in the skin was calculated [42]. The skin surface was then swabbed with ethanol five times and then flushed with water to eliminate excess drug from the surface, and the skin was dissected into small pieces. The tissue was further homogenised with ethanol and maintained at room temperature for 6 h. The extracted dispersions were vortexed at 4000 rpm for 15 min and collected using a 0.22 μm filter. Intradermal retention (*Q_m_*) per unit area was calculated according to Formula (3). All experiments were performed in triplicate.

(3) $$Q_{m}=\frac{\left(C_{m}\times V\right)}{A}$$

In the formula, *C_m_* is the extract concentration, *V* is the extract volume and *A* is the percutaneous penetration area.

### 3.6. In Vivo Pharmacodynamic Studies 

#### 3.6.1. Establishment of Psoriatic Animal Models

The mouse model of IMQ-induced psoriasis was created using KM mice [43,44], and the mice were randomly divided into 2 groups. In brief, with the exception of the normal group, IMQ cream (containing 3.125 mg of drug) was applied to the shaved dorsal skin of the mice every morning for 7 days. The group treated with IMQ was the model group. For normal-group mice equal amounts of white Vaseline were used once per day for 7 consecutive days.

#### 3.6.2. Psoriasis Area Severity Index (PASI) Evaluation

The PASI score was used to assess the severity of the redness, the thickness of the skin and the scaling elicited on the skin [45]. The scores range from 0 to 4 (0, none; 1, mild; 2, moderate; 3, marked; and 4, severe). The PASI evaluations were carried out every day for 7 days.

#### 3.6.3. Histopathologic Examination

The histopathological assessment of the skin samples was performed to evaluate the extent of pathological changes between the time of disease induction and after treatment. Animal skin samples were collected, fixed in 4% paraformaldehyde and deparaffinised. After deparaffinisation and rehydration, sections were subjected to haematoxylin and eosin (H&E) immersion staining and microscopic examination [46].

#### 3.6.4. Weight Ratio of Spleen to Body (Spleen/Body wt%)

The spleen is the largest part of the immune system of the human body. The spleen weight is an independent index of immune stimulation, with an elevated spleen/body weight ratio indicating that immune cells are proliferating in the spleen, which may reflect activation of the immune system [47,48,49]. Mouse spleens were separated after euthanization and quickly weighed to avoid measurement errors resulting from dehydration.

#### 3.6.5. The Levels of IL-17 and IL-6 Detection

At the end of the experiment, blood was harvested from the ophthalmic venous plexus, centrifuged at 4 °C, 10,000 rpm for 5 min to obtain the serum and analysed with mouse ELISA kits (Shanghai Yuanju Biotechnology Center, Shanghai, China).

## 4. Conclusions

Based on the preliminary studies above, it has been concluded that fatty acid vesicles offer potential benefits as carriers for delivering therapeutic molecules in the treatment of skin diseases. They are both cost-effective and therapeutically viable. They can fuse with the stratum corneum to enable the drug to enter the cellular interstitial space or to accumulate at the site of skin inflammation. Additionally, the skin’s high concentration of fatty acids can enhance the permeation of bioactive substances by interacting with the lipid matrix, thereby reducing skin diffusion resistance [27,28]. This phenomenon is crucial in the topical treatment of psoriasis and introduces a new option for transdermal drug delivery. Moreover, this discovery provides a valuable reference and analysis for future practical applications of transdermal drug delivery.

## Figures and Tables

**Table 1:** Results of Tre-FAV stability at 4 °C (*n* = 3).

Time/d	Size/nm	Zeta Potential/mv	EE% (Tre)
1	126.37 ± 1.290	−28.90 ± 1.920	84.26 ± 0.816
7	135.57 ± 1.361	−27.33 ± 0.651	83.10 ± 1.054
15	143.90 ± 1.453	−27.57 ± 0.569	81.83 ± 0.451
30	146.33 ± 0.709	−26.60 ± 0.608	80.77 ± 0.681

**Table 2:** Results of Tre-FAV stability at 25 °C (*n* = 3).

Time/d	Size/nm	Zeta Potential/mv	EE% (Tre)
1	121.22 ± 1.311	−26.71 ± 1.560	83.77 ± 0.656
7	136.87 ± 1.421	−26.23 ± 0.551	81.81 ± 1.334
15	144.50 ± 1.213	−26.11 ± 0.361	80.23 ± 0.321
30	157.31 ± 0.679	−25.16 ± 0.558	77.17 ± 0.881

## Data Availability

Data will be made available on request.
